# Pelvic Aggressive Angiomyxoma: Major Challenges in Diagnosis and Treatment

**DOI:** 10.7759/cureus.4419

**Published:** 2019-04-09

**Authors:** Roy Hajjar, Mohammed Alharthi, Carole Richard, François Gougeon, Rasmy Loungnarath

**Affiliations:** 1 Surgery, University of Montreal Health Centre, Montreal, CAN; 2 Pathology, University of Montreal Health Centre, Montreal, CAN

**Keywords:** aggressive angiomyxoma, pelvic tumors, perirectal tumor, luteinizing hormone-releasing hormone (lhrh) agonist

## Abstract

Aggressive pelvic angiomyxoma is a very rare mesenchymal tumor that is usually diagnosed in premenopausal female patients. The current mainly reported treatment is wide surgical excision. Other treatment options, such as radiotherapy and hormonal therapy, have been suggested as potential alternatives. A 61-year-old postmenopausal female patient presented with hematuria that led to the identification of a perirectal mass on abdominopelvic imaging. A 46-year-old female patient presented with a perineal mass of unknown etiology. Despite extensive investigations, the diagnosis could not be confirmed before surgical resection in both patients. Surgical excisions were performed and revealed the presence of an aggressive angiomyxoma with positive estrogen and progesterone tumoral receptors in both cases. Radiological and clinical recurrence was noted in one patient. Tumor regression was noted in this patient after treatment with a luteinizing hormone-releasing hormone (LHRH) agonist with long-term remission. The diagnosis of a perirectal aggressive angiomyxoma is an exceedingly rare event. Preoperative biopsy and pathological diagnosis are challenging and often yields poor results. Its slow growth and expression of hormonal receptors make noninvasive therapeutic strategies, such as radiotherapy, gonadotropin-releasing hormone agonists, or even watchful waiting, valid options in selected patients. Due to the lack of reported cases, the best treatment has yet to be elucidated.

## Introduction

Aggressive angiomyxoma (AA) is a rare mesenchymal tumor usually found in the pelvis of young female patients [1-2]. Steeper and Rosai reported the first documented cases in 1983 as locally infiltrative tumors with a local recurrence tendency [1]. Reported cases have mainly been described as invading the vulva, buttocks, or perineum [2]. Perirectal AA is an exceedingly rare, slow-growing neoplasm whose deep pelvic location poses serious challenges to its preoperative clinical and pathophysiological identification and whose best management has yet to be elucidated.

## Case presentation

Case 1

A 61-year-old female patient was evaluated at our university hospital for a perirectal tumor of unknown origin. Her past medical history included dyslipidemia and non-neoplastic postmenopausal vaginal bleeding. She had undergone a hysterectomy and bilateral salpingo-oophorectomy two years prior to the actual episode. Her medication included hormone replacement therapy (HRT). 

**Figure 1:**
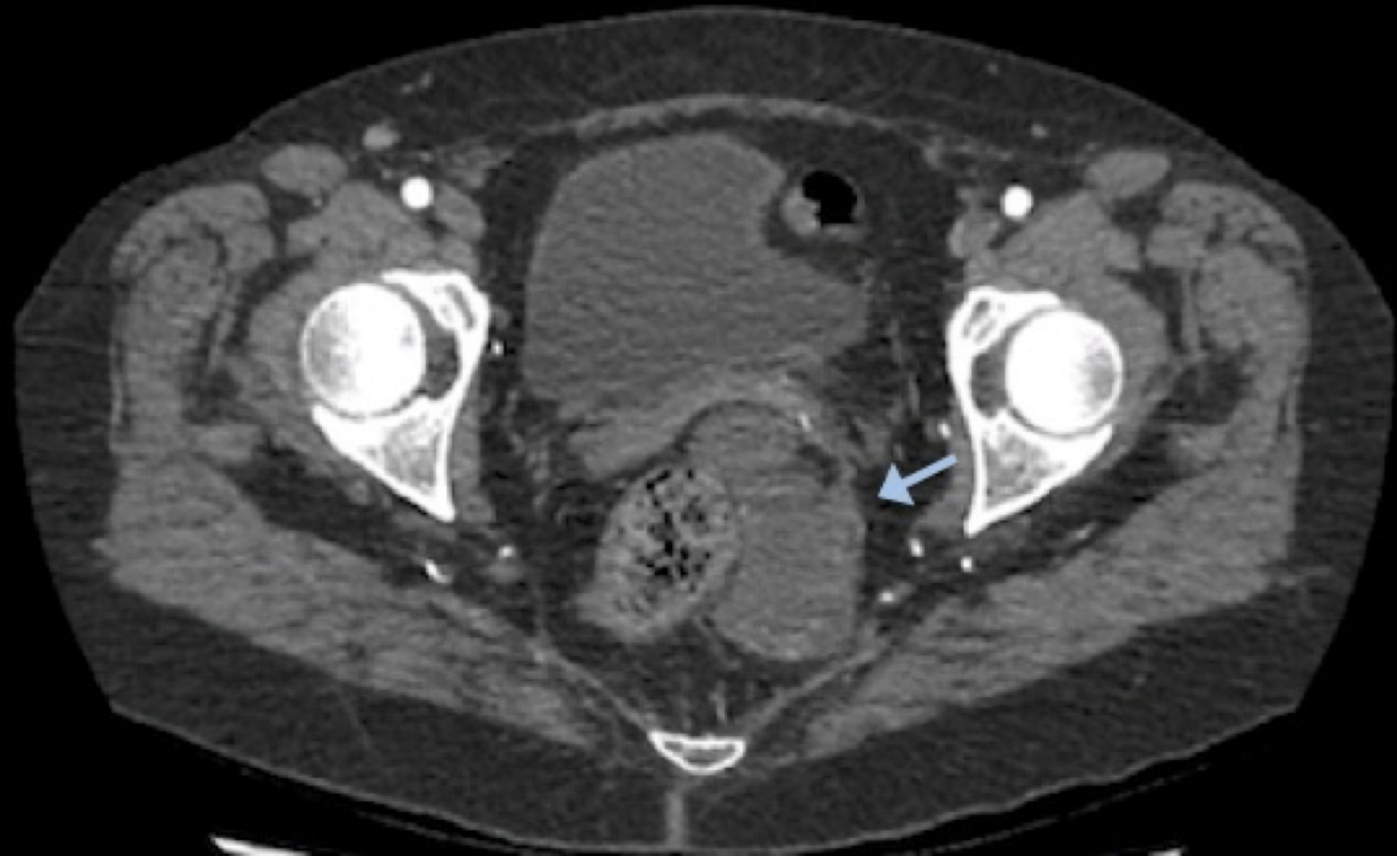
Abdominopelvic computed tomography (CT) scan revealing a left perirectal mass

The patient presented initially with macroscopic hematuria. A urological assessment, including a cystoscopy, did not reveal any identifiable cause for her complaint. An abdominopelvic computed tomography (CT) scan was performed as part of the investigation and showed a hypodense left perirectal mass with enhancing borders and ischiorectal extension (Figure 1). An abscess was initially suspected. The patient’s symptoms consisted of suprapubic pain for the past year and lower back pain during defecation, which did not support the infectious premise. Abdominal and vaginal examinations were normal. A rectal examination revealed a soft left extraluminal lump.

**Figure 2:**
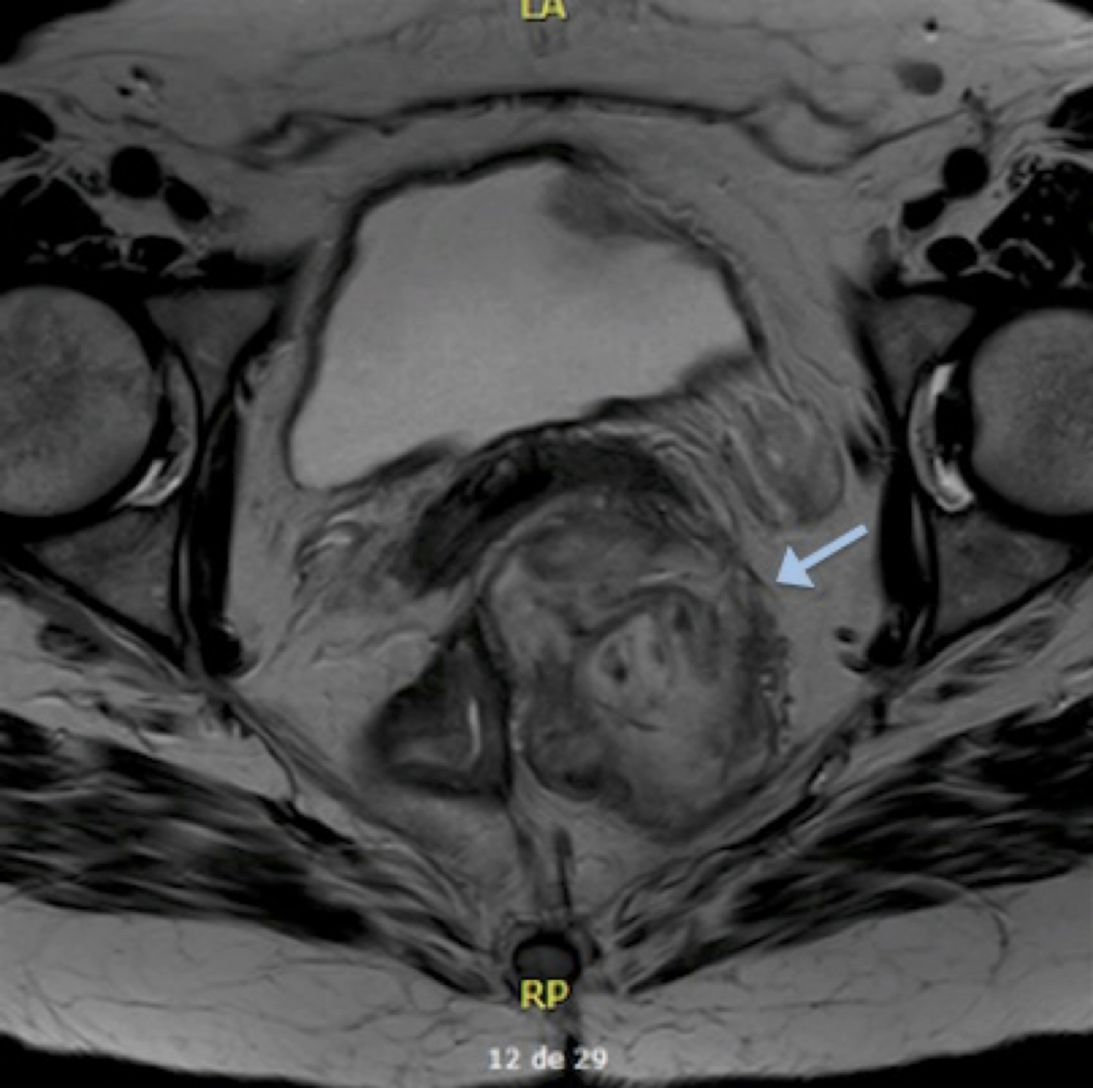
Pelvic magnetic resonance imaging Left perirectal mass pushing the rectum slightly to the right

**Figure 3:**
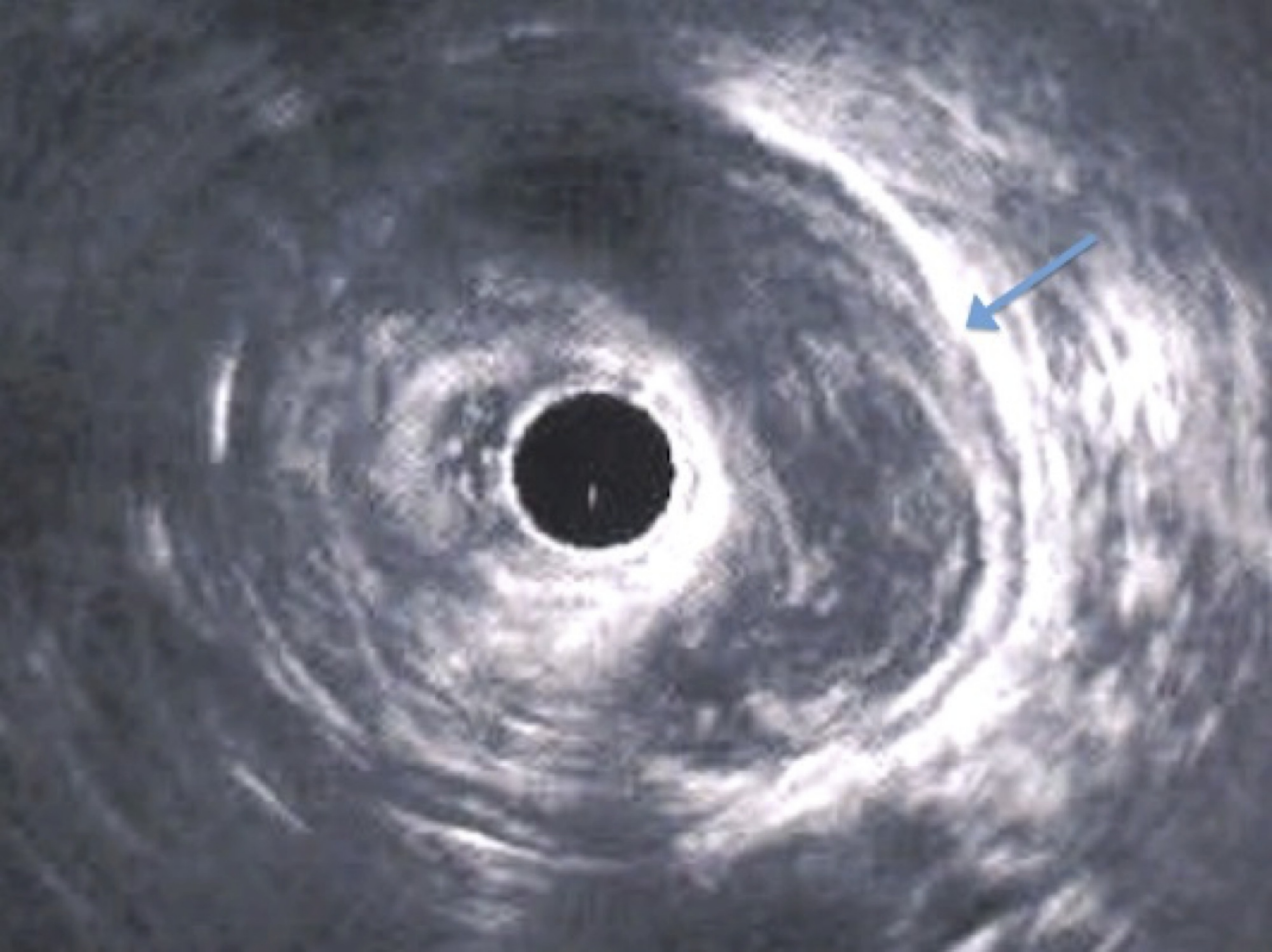
Transrectal ultrasonography Left nonspecific perirectal mass

Pelvic magnetic resonance imaging (MRI) revealed a left perirectal mass of 10.6 x 10.7 x 4.9 cm, which was in contact with the left posterolateral vaginal wall (Figure 2). Transrectal ultrasonography showed a nonspecific left perirectal mass (Figure 3).

A fine needle biopsy was performed but was inconclusive. A positron-emission tomography (PET) scan showed a mild hypermetabolic state in the mass, but it could not differentiate between a benign or malignant condition. No metastases were objectified. Our tumor board recommended a surgical resection, and the patient consequently underwent an open uncomplicated tumoral excision. The mass was not visible intraoperatively until the pouch of Douglas was opened. The rectum was left in place.

The pathological examination found a myxoid tumor without atypia or significant mitotic activity. Expression of estrogen (ER) and progesterone receptors (PR) was positive. Histological and immunohistochemical (IHC) features were consistent with an AA with positive microscopic margins.

The patient recovered uneventfully, except for a local pelvic abscess which was treated with antibiotics and percutaneous drainage. An abdominopelvic CT scan performed three months after the surgery showed no signs of recurrence.

Case 2

A 46-year-old healthy female patient was evaluated at our institution for a perirectal mass of unknown etiology. Her past medical history was unremarkable. The patient reported a perineal mass in the standing position. Her only other symptom consisted of mild occasional dysuria. Rectal and vaginal examinations were normal.

**Figure 4:**
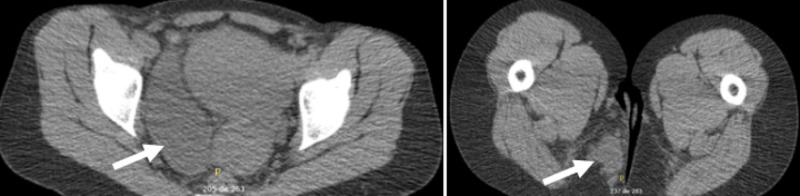
Abdominopelvic scan Right pelvic perirectal tumor

An endovaginal ultrasound performed one year before the present events to assess a known voluminous fibroma made no mention of a pelvic mass. An ultrasound of the perineal soft tissues found a hypoechogenic and heterogeneous mass of 4 x 5 cm beneath the paramedian region of the buttock. Endoscopic ultrasound showed a pelvic heterogenous mass that did not seem to originate from the rectal wall. A pelvic MRI and scan were performed and revealed a tubular structure of 13 x 7.5 x 3.5 cm in the right parametrium. It was centered around the right adnexal region, crossing the pelvic floor, reaching the ischiorectal fossae, and continuing as digital extensions in the soft tissue of the buttock (Figure 4). No invasion of the muscles, sphincters, or vaginal wall was noted. The possible suspected etiologies included an endometrioma, a parametrial cyst, a low-grade sarcoma, or another mesenchymal tumor. The absence of soft tissue infiltration ruled out an inflammatory cause. A PET scan showed a mild metabolism in the tumor, suggesting a cystic lesion. The diagnosis of a low-grade mesenchymal tumor could not, however, be excluded.

This unusual case was discussed at the institutional tumor board meeting and the decision was to proceed with surgical excision. The patient underwent an open surgical resection of the tumor. Due to a voluminous uterine fibroma that limited access to the pelvic cavity, an abdominal hysterectomy was performed. The pathological examination found a lesion with a proliferation of paucicellular spindle cells in a myxoid stroma and medium to thick-walled vessels of variable caliber. The stroma was without atypia. No mitoses or necrosis were identified. The histological aspect and immunohistochemical profile were consistent with the diagnosis of AA. The evaluation of the uterine specimen revealed two leiomyomas of 1.5 cm and 5.5 cm.

**Figure 5:**
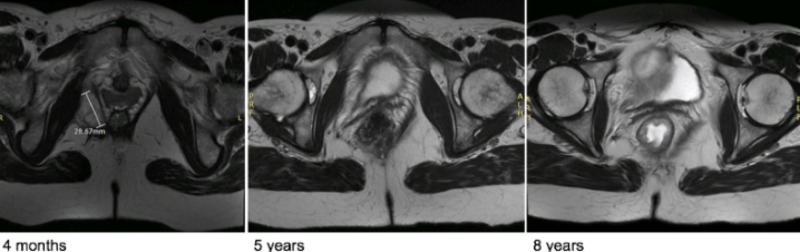
Pelvic MRI Postoperative evolution

The patient’s evolution was satisfactory one month after the surgery with no symptoms suggesting a possible recurrence. An MRI performed four months after the procedure showed a right perirectal longitudinal tissular density with a length of 6 cm (Figure 5). Imaging could not confirm if it was a recurrent mass, a surgical scar, or an incomplete excision. Due to a positive ER and PR, the decision of the tumor board was to initiate tamoxifen. One month later, the patient reported the feeling of a perineal mass, similar to the one felt prior to surgery. Another MRI was performed and showed a progression of the tissular density that extended upward to a length of 10 cm. The case was further discussed with the Gynecology-Oncology team and the joint decision was to start a luteinizing hormone-releasing hormone (LHRH) agonist.

Three months later, a repeat MRI found a considerable improvement with a sharp decline in the lesion’s extent, now measuring 3.1 x 2.3 x 1.6 cm. One year after the surgical excision, the lesion’s dimensions had further decreased to a length of 2.6 cm. An MRI 32 months after the surgery found a small residual centimetric lesion. After four years, an MRI revealed a thin image of residual fibrocicatricial tissue. After five and eight years, respectively, a repeat MRI showed no signs of recurrence and stability of a small residual local scar (Figure 5).

## Discussion

Herein, we present two very rare cases of perirectal AA incidentally diagnosed in a postmenopausal woman and in a premenopausal woman.

The majority of AA cases in women are diagnosed in premenopausal patients [3]. Reported cases are commonly described as incidental findings or insidious slow-growing vulvar or perineal masses that sometimes go unnoticed or unrecognized for years before a proper and correct diagnosis is made [3-4].

Although much scarcer, some cases of AA have been reported in male patients as paratesticular masses [5-6]. In female patients, this type of tumor has been reported to display ER and PR positivity, which is believed to play a role in tumoral evolution before menopause [2, 4]. Data on AA in males is lacking to allow a proper understanding of the clinical evolution and the exact influence of hormones on tumor growth.

AA is usually diagnosed in premenopausal patients. The positivity of tumoral hormonal receptors may influence its development in women of childbearing age. The diagnosis of AA in our first postmenopausal patient is very unusual. One may wonder if the HRT the patient was receiving might have influenced the tumor’s growth.

Macroscopically, AA has been described as having both smooth and adherent margins that infiltrate the host’s tissues [2, 4]. The histopathological characteristics consist of a population of hypocellular spindle cells sparsely spread in a loose myxoid matrix with collagen bundles [2-4]. AA has been reported to have a recurrence rate of 41% and is described as having generally the same histological traits when recurring [4, 7]. The tumoral stroma tends to display many blood vessels of various calibers [2, 4]. The tumoral vessels in our patient were thus assessed through imaging studies to determine if preoperative angioembolization was required. It is also worth noting that histological sampling before surgery is particularly difficult with deep perirectal tumors, as shown in our patient, with whom biopsies were inconclusive. Also worth mentioning is that both pathological evaluations in our cases were reviewed by specialized soft tissue pathologists.

The best treatment for AA remains unknown. Wide surgical excision is the main reported therapeutic approach [4, 8-10]. Achieving negative microscopic margins seems an obvious principle, but a literature search failed to prove that negative resection margins prevent recurrence. The extent of surgical excision has thus to be weighed against its risks, especially in young patients of childbearing age. Although the rectum was left in place in both patients, surgical resection of the tumor may be challenging due to the close proximity of the mass to the rectal wall. If available, robotic surgery may be a potentially convenient approach in a narrow pelvic cavity.

Radiation therapy is a potential option that has been described as an alternative to extensive resection in advanced disease or as an adjuvant treatment for recurrent tumors [4, 7-8]. Its use as the sole therapeutic approach for AA, which has a low mitotic rate, is controversial, however [4]. Some authors have suggested using gonadotropin-releasing hormone agonists as a neoadjuvant treatment to downsize the tumor prior to surgery or as an adjuvant treatment to prevent recurrence in tumors with positive ER and PR [7]. The use of this hormonal agonist in a recurrent case led to a complete remission of the disease, thus avoiding aggressive surgeries [11]. Data on the effectiveness of this option is lacking, especially since not all cases are reported in women and the fact that postmenopausal patients may not display the same tumoral pathophysiological features as premenopausal patients [4, 8-9]. Nonetheless, it showed a significant benefit in our patient since the recurrent tumor responded completely to an LHRH agonist. Whether LHRH agonists could be useful after menopause has yet to be elucidated. In addition, one might wonder if the sole use of an LHRH agonist could have induced complete regression of the initial tumor. On another note, watchful waiting has been suggested as a justifiable option in some cases of asymptomatic tumors where a surgical approach poses more risks than benefits [4, 9].

Follow-up after surgery includes clinical and radiological examinations [4]. However, more cases are required to validate the modalities and frequency of postoperative follow-up.

## Conclusions

In conclusion, surgical resection of AA seems to be a valid option, but the local and slow-growing features of this tumor make other noninvasive approaches reasonable in some patients. Nonetheless, note that the scarcity of data on the specific pathophysiology of AA and the inability to accurately predict its exact evolution pose serious challenges to the decision-making process. The perirectal location of AA poses an additional challenge to its diagnosis and treatment, as a surgical intervention may put several major anatomic pelvic structures at risk, especially since the role of surgical excision has been repeatedly challenged in favor of noninvasive alternatives.
